# MicroRNA-326 attenuates immune escape and prevents metastasis in lung adenocarcinoma by targeting PD-L1 and B7-H3

**DOI:** 10.1038/s41420-021-00527-8

**Published:** 2021-06-15

**Authors:** Lijuan Shao, Qian He, Jingbo Wang, Fei He, Shengcheng Lin, Liujing Wu, Yubiao Gao, Wei Ma, Jun Dong, Xiaofei Yang, Furong Li

**Affiliations:** 1 Translational Medicine Collaborative Innovation Center, Shenzhen People’s Hospital (The Second Clinical Medical College, Jinan University; The First Affifiliated Hospital, Southern University of Science and Technology), Shenzhen, 518020 China; 2 Shenzhen key laboratory of stem cell research and clinical transformation, Shenzhen, 518020 China; 3 Integrated Chinese and Western Medicine Postdoctoral Research Station, Jinan University, Guangzhou, 510632 China; 4 National Cancer Center/National Clinical Research Center for Cancer/Cancer Hospital & Shenzhen Hospital, Chinese Academy of Medical Sciences and PeKing Union Medical College, Shenzhen, 518116 China

**Keywords:** Immunoediting, Immunotherapy

## Abstract

Tumor-infiltrating T cells are highly expressive of inhibitory receptor/immune checkpoint molecules that bind to ligand expressed by tumor cells and antigen-presenting cells, and eventually lead to T cell dysfunction. It is a hot topic to restore T cell function by targeting immune checkpoint. In recent years, immunotherapy of blocking immune checkpoint and its receptor, such as PD-L1/PD-1 targeted therapy, has made effective progress, which brings hope for patients with advanced malignant tumor. However, only a few patients benefit from directly targeting these checkpoints or their receptors by small compounds or antibodies. Since the complexity of the regulation of immune checkpoints in tumor cells, further research is needed to identify the novel endogenous regulators of immune checkpoints which can help for developing effective drug target to improve the effect of immunotherapy. Here, we verified that microRNA-326 (miR-326) repressed the gene expression of immune checkpoint molecules PD-L1 and B7-H3 in lung adenocarcinoma (LUAD). We detected that the expression of miR-326 in LUAD tissue was negatively correlated with PD-L1/B7-H3. The repression of PD-L1 and B7-H3 expression through miR-326 overexpression leads to the modification the cytokine profile of CD8^+^ T cells and decreased migration capability of tumor cells. Meanwhile, the downregulation of miR-326 promoted tumor cell migration. Moreover, blocking PD-L1 and B7-H3 attenuated the tumor-promoting effect induced by miR-326 inhibitor. In tumor-bearing mice, the infiltration of CD8^+^ T cells was significantly increased and the expression of TNF-α, and IFN-γ was significantly enhanced which contributed to tumor progression after miR-326 overexpression. Collectively, miR-326 restrained tumor progression by downregulating PD-L1 and B7-H3 expression and increasing T cell cytotoxic function in LUAD. Our findings revealed a novel perspective on the complex regulation of immune checkpoint molecules. A new strategy of using miR-326 in tumor immunotherapy is proposed.

## Introduction

Lung cancer is the most frequent malignant tumor in the world [1]. Most patients are in the advanced stages when diagnosed [2]. Metastasis is the primary cause of death. Tumor immunotherapy has achieved rapid development and become one of the important strategies for advanced malignant tumors since the application of the first programmed cell death protein 1 (PD-1) antibody Opdivo [3, 4]. Antibodies that block inhibitory immune checkpoints, such as PD-1, programmed cell death ligand 1 (PD-L1), enhance the killing capacity of T cells against cancer cells, have achieved significant therapeutic effects in cancers, especially lung cancer and melanoma [4–6]. PD-1/PD-L1 immunotherapy effectively prolongs the survival time of patients with advanced non-small cell lung cancer (NSCLC). But not all patients are sensitive to immunotherapy. More research on the regulatory mechanism of immune checkpoint molecules is required for precise targeted therapy.

In this study, we demonstrated that Hsa-miRNA-326 (miRNA-326) downregulates the expression of PD-L1 and B7-H3 in B7 family members and thus prevents tumor immune escape. PD-L1, also known as B7-H1 and CD274, is continuously expressed in many solid tumors, such as lung cancer, ovarian cancer, and renal cell carcinoma [7, 8]. PD-L1 interact with PD-1 on T cells to make them lose their killing ability and tumors escape the surveillance and killing of the immune system, which promote tumor progression [7, 9, 10]. B7-H3, also called CD276, is overexpressed in many malignant tumors and closely related to the increased metastasis, recurrence, resistance to therapy, and poor prognosis of malignant tumors [11–13]. High expression of B7-H3 were proved in 74% of NSCLC [14] and showed positively associated with poor prognosis [11, 15]. B7-H3 repressed T cell proliferation and attenuated activity of T cells, thus promotes cancer cell immune escape [16–18].

MicroRNAs (miRNAs) are noncoding RNAs that repress gene expression by directly binding to the 3′ untranslated region (UTR) of target genes. Aberrant miRNA expression is closely related to the occurrence and progression of human cancers [19]. Many miRNAs have been reported as diagnostic biomarkers in cancers [20, 21]. In recent years, emerging researches have focused on the regulatory effect of miRNAs on tumor immune regulation. For instance, miR-200, miR-155, miR-138-5p, and miRNA-873 regulate PD-L1 expression; miR-33a, miR-155, miR-873, and miR-200 regulate checkpoints, such as PD-1 and CTLA-4 [22–26]. MiR-326 is a tumor-suppressing miRNA and is downregulated in various tumors, including lung cancer, gastric cancer, endometrial cancer, and breast cancer [27]. MiR-326 participates in tumor cell invasion, metastasis, drug resistance, proliferation, and apoptosis [27–29]. The upregulated expression of miR-326 limits tumor metastasis by targeting miR-326 reverse chemoresistance in human lung adenocarcinoma (LUAD) cells [30]. However, its regulatory mechanism in tumor is largely unknown, especially in tumor-associated immunosuppression.

In this study, we identified the direct regulation of PD-L1 and B7-H3 expression by miR-326 and the subsequent suppression of CD8^+^ T cells in the tumor microenvironment and highlighted a new potential therapeutic target to overcome immune evasion by tumor cells.

## Results

### miR-326 directly binds to 3′ UTRs of *PD-L1* and *B7-H3*

**Fig. 1:**
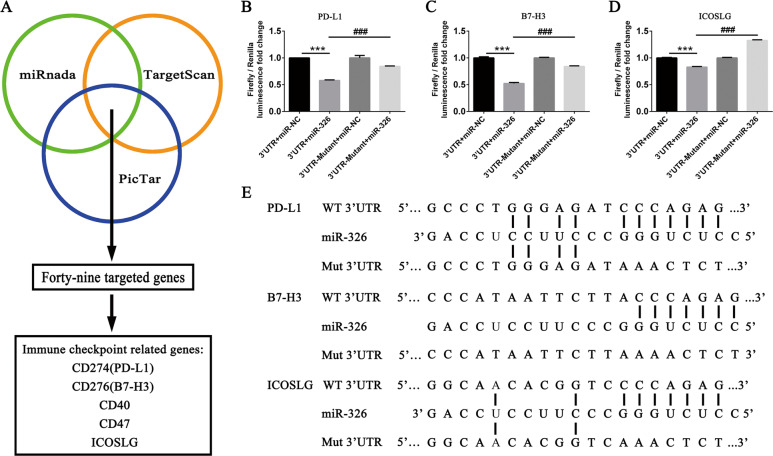
PD-L1, ICOSLG, and B7-H3 are the direct target genes of miR-326. **A** Software (miRBase, PicTar, and TargetScan) prediction of the miR-326-targeted genes. **B**–**D** 293T cells were transfected with luciferase reporter plasmid containing PD-L1/B7-H3/ICOSLG wild-type or mutant-type 3′ UTR and miR-NC (*n* = 3). **E** Schematic diagram of the binding sites between miR-326 and the target genes (PD-L1, B7-H3, and ICOSLG) and their mutant sequence. All data were presented as mean ± SEM. Comparisons between groups for statistical significance were performed with two-way ANOVA with Tukey’s post hoc test (**B**–**D**). ***P < 0.001 versus 3′ UTR + miR-NC. ^###^*P* < 0.001 versus 3′ UTR + miR-326.

Aberrantly expressed miR-326 participates in malignant progression of LUAD [31]. Nevertheless, how miR-326 regulates tumor immunity remains unclear. We utilized miRBase, PicTar, and TargetScan software to make prediction of miR-326 targeted genes and focused on the immune checkpoint-related genes. As shown in Fig. 1A, *PD-L1*, *B7-H3,* and *ICOSLG* genes in B7 family were supposed to be targeted by miR-326. We performed luciferase reporter assay to verify the direct binding of miR-326 to the putative targeted genes determined by bioinformatics target prediction analysis. Plasmid with the wild-type (WT) or mutant type (Mut) 3′ UTR of the targeted genes (PD-L1, ICOSLG, and B7-H3), miR-NC mimics, and miR-326 mimics were transfected into 293T cells. Obviously, miR-326 repressed wild-type luciferase reporter activity other than the mutant group (Fig. 1B–D). This result suggests that miR-326 directly binds to the 3′ UTRs of *PD-L1*, *B7-H3*, and *ICOSLG*, and might repress the expression of these genes. The predicted binding sites and mutant sequence are shown in Fig. 1E.

### MiR-326 is downregulated in LUAD and negatively related to PD-L1 and B7-H3

**Fig. 2:**
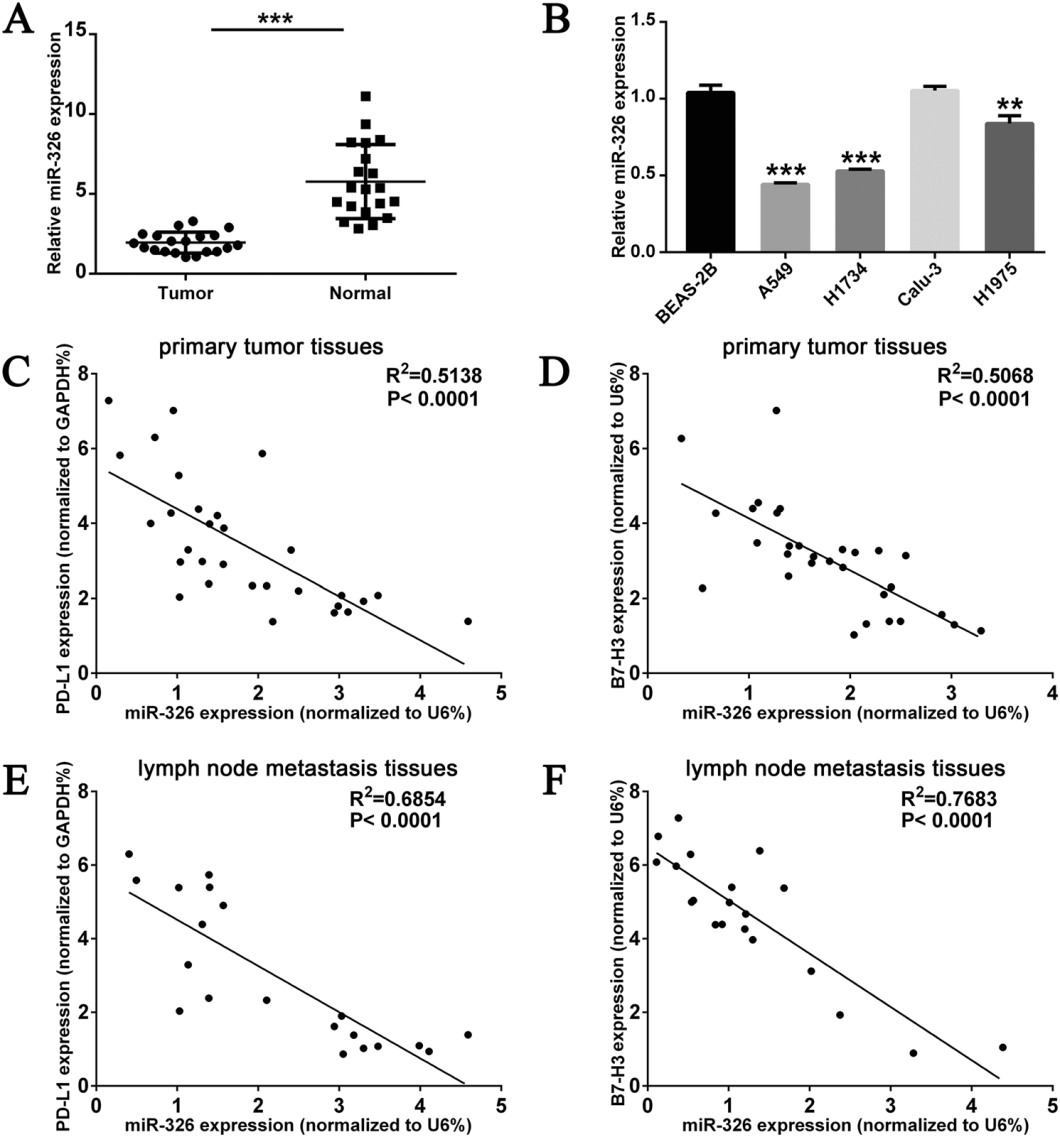
MiR-326 is downregulated in LUAD and inversely correlated with PD-L1 and B7-H3. **A** Transcription level of miR-326 in 20 matched LUAD tissues (tumor, *n* = 20) and adjacent normal tissues (normal *n* = 20) by qRT-PCR and normalized by an endogenous control U6. **B** Transcription level of miR-326 in normal lung epithelial cell line BEAS-2B and LUAD cell lines A549, H1734, Calu-3, and H1975 (*n* = 3). Dot plot of the concordant miR-326 and PD-L1 (**C**), B7-H3 (**D**) RNA levels in LUAD primary tumor tissues (*n* = 30). Dot plot of the concordant miR-326 and PD-L1 (**E**), B7-H3 (**F**) RNA levels in the lymph node metastasis tissues of LUAD (*n* = 20). All data were presented as mean ± SEM. Comparisons between groups for statistical significance were performed with Student’s *t* test (**A**, **B**). Spearman’s rank correlation coefficient was used to measure the association between miR-326 and PD-L1 (**C**, **E**) or B7-H3 (**D**, **F**). **P < 0.01, ***P < 0.001 versus tumor or BEAS-2B.

MiR-326 quantitation in LUAD tissues and adjacent normal tissues was accomplished by qRT-PCR. Consistent with previous reports and analysis from public datasets (Supplementary Fig. 1A), miR-326 had lower expression in LUAD tissues than adjacent normal tissues (Fig. 2A). Meanwhile, miR-326 expressed lower in LUAD cell lines than normal lung epithelial cell line BEAS-2B (Fig. 2B). The relationship of miR-326 and PD-L1/B7-H3 expression in LUAD tumor tissues were explored by qRT-PCR and Spearman’s rank-order correlation analysis. As shown in Fig. 2C, D, miR-326 is negatively expressed compared with PD-L1 and B7-H3 in tumor tissues. What is more, miR-326 and PD-L1/B7-H3 showed a higher negative correlation in lymph node metastasis than in in situ tissues (Fig. 2E, F). This result implies a possible correlation related to tumor metastasis. However, the expression of miR-326 has no substantial relevance to ICOSLG in primary tumor and lymph node metastatic tissues (Supplementary Fig. 1B, C). Overall, these findings prompt that miR-326 probably mediates the immunoreaction in LUAD through regulating the expression of *PD-L1* and *B7-H3*.

### MiR-326 attenuates PD-L1 and B7-H3 expression in LUAD cells

**Fig. 3:**
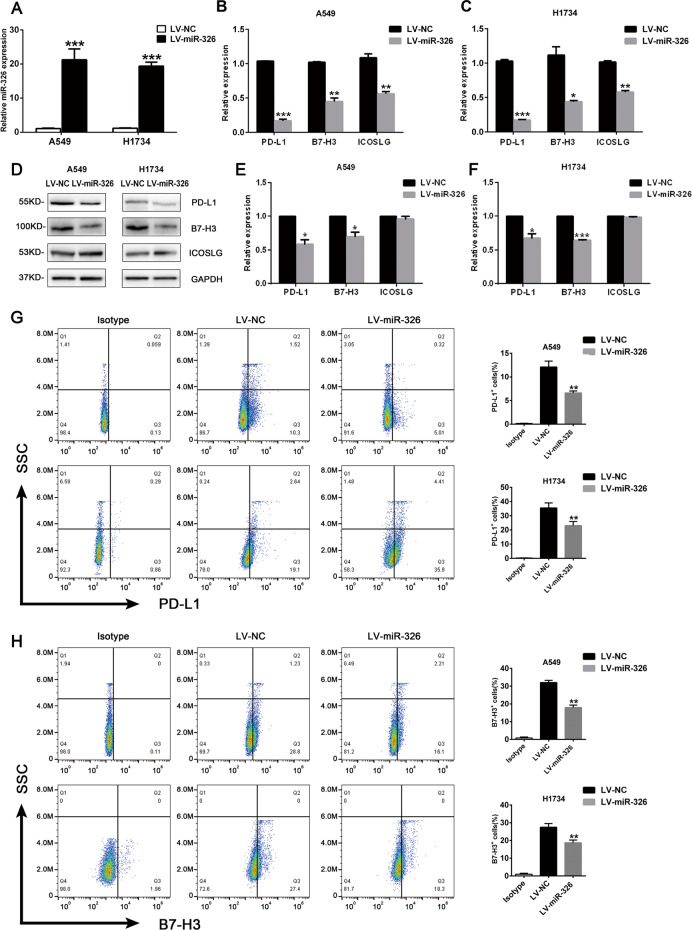
MiR-326 repressed PD-L1 and B7-H3 expression in LUAD cells. Lentivirus was used to overexpress miR-326 in A549 and H1734 cells. **A** Relative expression of miR-326 72 h after lentivirus infection was quantified by qRT-PCR (*n* = 3). **B**, **C** Relative expression of PD-L1/B7-H3 in miR-326 overexpressed A549 cells and H1734 cells was quantified by qRT-PCR (*n* = 3). **D** Protein levels of PD-L1 and B7-H3 after miR-326 overexpression were determined by western blots. **E**, **F** Statistical histogram of the western blots (*n* = 3). PD-L1 (**G**) and B7-H3 (**H**) expression on tumor cells was detected by flow cytometry (*n* = 3). All data were presented as mean ± SEM. Comparisons between groups for statistical significance were performed with Student’s *t* test. *P < 0.05, **P < 0.01, ***P < 0.001 versus LV-NC.

We performed lentivirus infection to overexpress miR-326 in LUAD cell lines A549 and H1734 to confirm the direct regulation of miR-326 on PD-L1 and B7-H3. Figure 3A shows the efficiency of overexpression. As testified by qRT-PCR and western blot in Fig. 3B–F, enforced miR-326 decreased the expression of PD-L1 and B7-H3. However, overexpressed miR-326 only leads to considerable recession of ICOSLG at the RNA level but not in the protein level (Fig. 3B–F). In addition, flow cytometry analysis also revealed that PD-L1 and B7-H3 on A549 and H1734 cell lines was remarkably downregulated (Fig. 3G, H). Conversely, PD-L1 and B7-H3 expression increased when miR-326 was downregulated through miR-326 inhibitor in Calu-3 and H1975 cell lines (Supplementary Fig. 2). Overall, the results indicate that miR-326 repressed PD-L1 and B7-H3 expression in LUAD.

### MiR-326 modifies the cytokine profile of CD8^+^ T cells and represses tumor cell migration in vitro

**Fig. 4:**
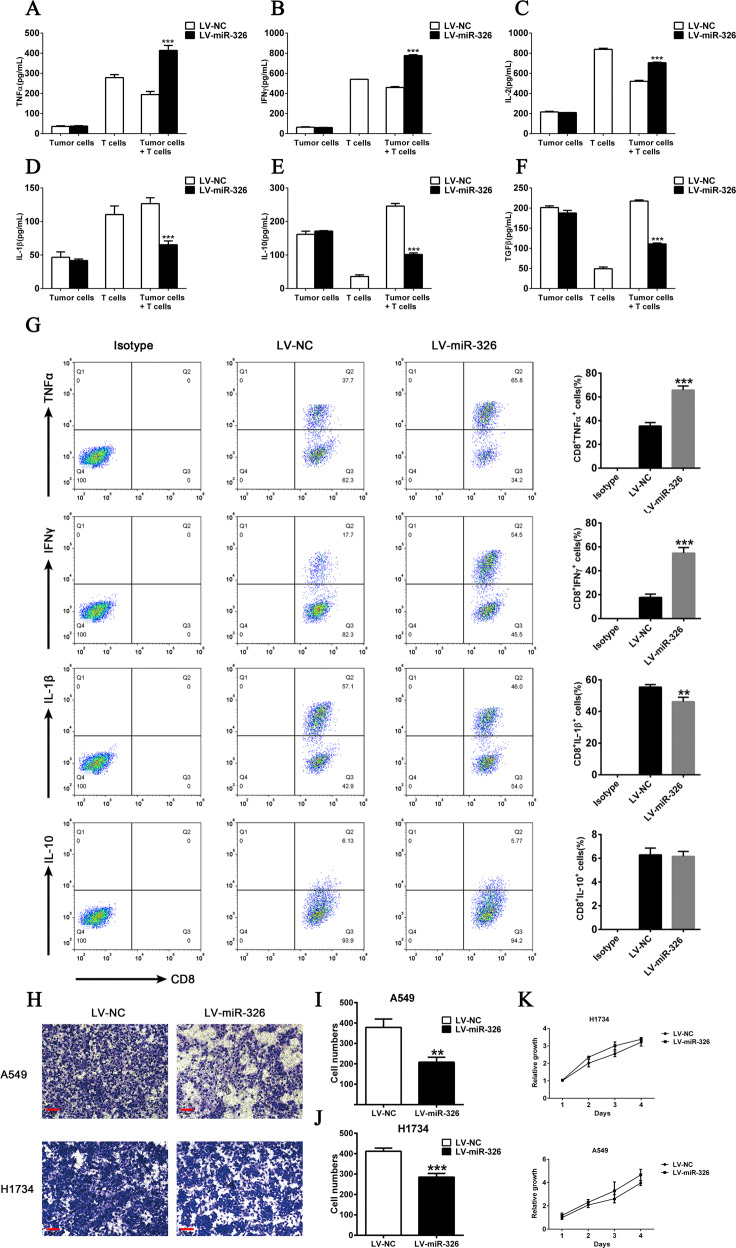
MiR-326 in tumor cells regulates CD8^+^ T phenotype and tumor cell migration ability. **A**–**F** T cells were co-cultured with miR-NC- or miR-326-overexpressed H1734 cells. Cytokines in the supernatant were measured by ELISA (*n* = 3). **G** TNF-α, IFN-γ, IL-10, and IL-1β expression of CD8+ T cells after miR-326 overexpression by flow cytometry (*n* = 3). **H** Transwell assay of control and miR-326-overexpressed cells. **I**, **J** Histogram of the number of migrated cells (*n* = 3). **K** MTS analysis of the proliferation of miR-NC- or miR-326-overexpressed cells. All data were presented as mean ± SEM. Comparisons between groups for statistical significance were performed with two-way ANOVA with Tukey’s post hoc test (**A**–**F**, **K**) or Student’s *t* test (**G**, **I**, **J**). **P < 0.01, ***P < 0.001 versus LV-NC. Scale bar, 200 µM.

PD-L1 and B7-H3 were supposed to cause T cell dysfunction and accelerate tumor progression, and miR-326 was suggested to repressed PD-L1 and B7-H3 expression. Thus, we intended to determine whether miR-326 affects T cell function by modulating PD-L1 or B7-H3. We constructed a co-culture system of tumor cell and T cells induced by peripheral blood. Then, we detected changes in T cell function. As shown in Fig. 4A–F, TNF-α, IFN-γ, and IL-2 increasingly secreted in the supernatant of miR-326 overexpressed cells. Meanwhile, IL-1β, IL-10, and TGF-β were downregulated. To further verify whether the change of cytokines’ profile was derived from T cells, we isolated CD8^+^ T cells and examine their cytokine expression by flow cytometry. As shown in Fig. 4G, the TNF-α and IFN-γ positive population in CD8^+^ T cells was significantly increased after miR-326 overexpression, while the IL-10 positive population was not changed and the IL-1β positive population was slightly reduced. Moreover, miR-326 and PD-L1/B7-H3 had a stronger negative correlation in lymph node metastasis than in situ, which implied that miR-326 might be related to metastasis. Therefore, we performed transwell assay for the cells in miR-326 overexpressed cells and found that enforced miR-326 substantially inhibited cell migration (Fig. 4H–J). Meanwhile, MTS assay analysis showed that miR-326 overexpression did not significantly affect the cell proliferation (Fig. 4K), which demonstrated that miR-326 probably promotes the tumor metastasis.

### MiR-326 regulating the cytokine profile of CD8^+^ T cells and tumor cell migration is PD-L1/B7-H3-dependent

**Fig. 5:**
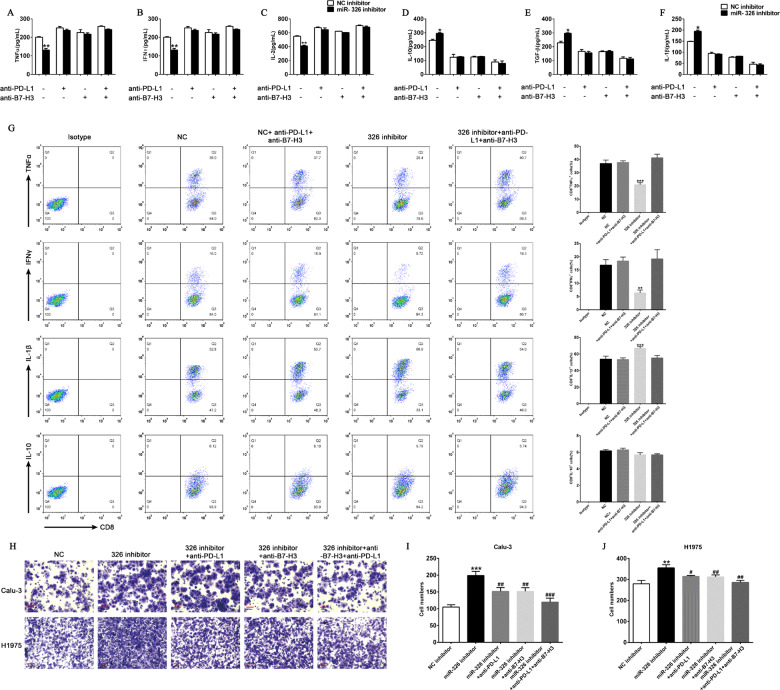
Blockade of PD-L1 and B7-H3 markedly abrogates the aggressive tumor behavior caused by miR-326 downregulation. **A**–**F** T cells were co-cultured with miR-NC- or miR-326-overexpressed H1975 cells, and PD-L1/B7-H3 blocking antibody was added. Cytokines in the supernatant were measured by ELISA (*n* = 3). **G** TNF-α, IFN-γ, IL-10, and IL-1β expression of CD8+ T cells after miR-326 overexpression and/or PD-L1/B7-H3 antibody incubation by flow cytometry (*n* = 3). **H** Transwell assay of control and miR-326-overexpressed cells, and PD-L1/B7-H3 blocking antibody was added. **I**, **J** Histogram of the number of migrated cells (*n* = 3). All data were presented as mean ± SEM. Comparisons between groups for statistical significance were performed with one-way (**I**, **J**) or two-way (**A**–**G**) ANOVA with Tukey’s post hoc test. *P < 0.05, **P < 0.01, ***P < 0.001 versus NC inhibitor. ^##^*P* < 0.01, ^###^*P* < 0.001 versus miR-326 inhibitor. Scale bar, 200 µM.

We speculate that miR-326 inhibits T cell function through regulating PD-L1 and B7-H3 expression. First, we repressed miR-326 expression in H1975 cell lines and then co-cultured the cells with T cells. As expected, the downregulation of miR-326 in H1975 cells leads to the decrease of TNF-α, IFN-γ, and IL-2 secretion and the increase of IL-1β, IL-10, and TGF-β secretion (Fig. 5A–F). Moreover, administration of PD-L1 and/or B7-H3 antibody considerably reverted the level of cytokine secretion (Fig. 5A–F). Consistently, the downregulation of TNF-α and IFN-γ positive population and upregulation of and IL-1β positive population in CD8^+^ T cells by miR-326 inhibitor were also rescued by PD-L1 and B7-H3 antibody incubation (Fig. 5G). These findings suggest that miR-326 affects the function of CD8^+^ T cell secretion by interfering PD-L1 and B7-H3 function. Furthermore, downregulated miR-326 markedly enhanced the migration ability of tumor cells, and blocking PD-L1 or B7-H3 could reverse the effect caused by repressed miR-326 (Fig. 5H–J). Overall, miR-326 prevents tumor cell migration through repressing PD-L1 and B7-H3.

### Overexpression of miR-326 enhances antitumor efficacy by blocking PD-L1 and B7-H3 in vivo

**Fig. 6:**
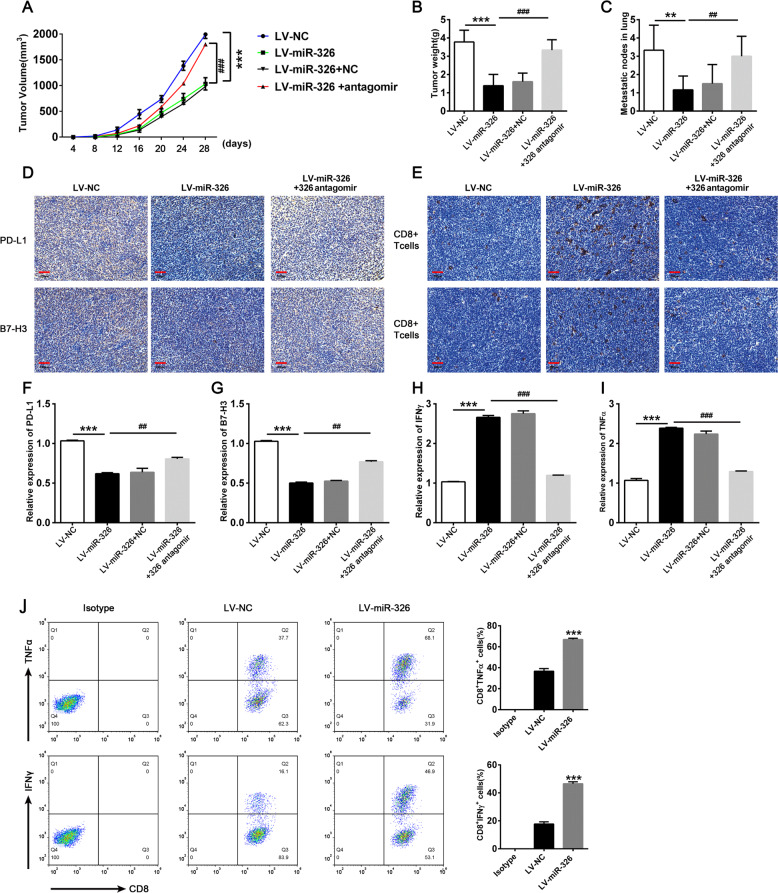
Overexpression of miR-326 enhances TNF-α and IFN-γ expression of infiltrated CD8^+^ T cells and prevents metastasis by targeting PD-L1 and B7-H3 on cancer cells. 5 × 10^5^ miR-326 overexpressed LLC cells or empty vector control LLC cells were subcutaneously injected into C57BL/6 mice. MiR-326 antagomir or NC antagomir were intratumor injected when the tumor diameter reached 0.5 cm. The mice were sacrificed 28 days after inoculation. **A** Tumor volume (*n* = 7). **B** Tumor weight (*n* = 6). **C** Pulmonary metastasis nodule calculated by hematoxylin–eosin staining. (*n* = 6) **D**, **E** PD-L1, B7-H3 expression, and CD8^+^ T cells infiltration were determined by IHC staining in tumor tissues. **F**–**I** PD-L1, B7-H3, TNF-α, and IFN-γ were detected by qRT-PCR in tumor sites (*n* = 3). **J** TNF-α and IFN-γ expression of infiltrated CD8^+^ T cells by flow cytometry (*n* = 3). All data were presented as mean ± SEM. Comparisons between groups for statistical significance were performed with one-way (**B**, **C** and **F**–**I**) or two-way (**A**) ANOVA with Tukey’s post hoc test or Student’s *t* test (**J**). *P < 0.05, ***P < 0.001 versus LV-NC. ^#^*P* < 0.05, ^##^*P* < 0.01, ^###^*P* < 0.001 versus LV-miR-326. Scale bar, 200 µM.

C57BL/6 mice were challenged with stable miR-326 overexpression (LV-miR-326) or vector negative control (LV-NC) lung cancer cells (LLCs). MiR-326 antagomir or negative control (10 nmol) was injected into the tumor every 4 days for 28 days to further confirm the antitumor effect of miR-326. The tumor growth curve and tumor weight are shown in Fig. 6A, B. MiR-326 overexpression substantially decreased tumor volume and tumor weight in mice indicating that miR-326 overexpression can restrain tumor growth. Moreover, miR-326 overexpression tumor showed less pulmonary metastasis nodule than negative control (Fig. 6C). In addition, the depletion of miR-326 through miR-326-specific antagomir restored the antitumor effect of miR-326 (Fig. 6A–C). Tumor tissues were taken for IHC staining after the mice were sacrificed to detect PD-L1 and B7-H3 expression. As expected, PD-L1 and B7-H3 were lower in the miR-326 overexpression group than that in miR-NC group (Fig. 6D). Then, we performed IHC staining for CD8^+^ T cells infiltrated in tumor sites. Obviously, miR-326 overexpression tumor tissues showed more CD8^+^ T cells infiltration (Fig. 6E). Then, we extracted RNA from tumor tissues and performed qRT-PCR to detect PD-L1 and B7-H3 and cytokine expression. Consistent with the results above, the levels of PD-L1 and B7-H3 was reduced (Fig. 6F, G) when miR-326 was overexpressed, while the levels of TNF-α and IFN-γ increased (Fig. 6H, I). Furthermore, intratumor injection of miR-326 antagomir substantially reverted the phenotype caused by miR-326 (Fig. 6F–I). Additionally, the expression of TNF-α and IFN-γ was also upregulated in infiltrated CD8^+^ T cells after miR-326 overexpression (Fig. 6J). Collectively, miR-326 modifies the cytokine profile of CD8^+^ T cells and repressed tumor progression by regulating PD-L1 and B7-H3 expression.

## Discussion

Immunotherapy has made great progress in recent years. Blocking immune checkpoints is one of the most promising strategies for activating antitumor immunity [32, 33]. In recent years, advances in targeted therapy for immune checkpoints, such as PD-L1/PD-1, brings hope for patients with advanced stages [8, 34]. However, not all patients benefit from immunotherapy. The heterogeneous level of PD-L1 in patients indicates that PD-L1 expression is affected by many regulatory mechanisms. Even some PD-L1-positive patients are refractory for treatment. The immune-related gene expression is under control of many regulators with diverse regulatory mechanisms [35]. Thus, exploring the upstream modulators of immune checkpoint molecules can broaden our understanding to develop novel drug targets.

In recent years, miRNAs have attracted much attention as a class of important gene regulatory factors. These noncoding RNAs repress gene expression and participate in pathological processes [20]. Some miRNAs are also involved in immune regulation [36, 37]. MiR-326 is involved in the progress of various tumors, including lung cancer, and acts as an inhibitory factor [21, 38, 39]. However, the regulatory effect of miR-326 on immune checkpoint has been rarely explored.

In this study, we utilized forecasting software to predict the targeted genes related to immunotherapy. Among the 49 genes, we focused on PD-L1, ICOSLG, and B7-H3 (Fig. 1A). Luciferase reporter assay proved that miR-326 directly bind to the 3′ UTRs of PD-L1, ICOSLG, and B7-H3 (Fig. 1B–D).

PD-L1, ICOSLG, and B7-H3 are all members of B7 family. B7 family molecules are the immunoglobulin superfamily members that can provide stimulation signals to enhance and maintain T cell immune responses and also produce inhibitory signals to limit and weaken T cell immune responses. Dysregulated expression of B7 family will lead to T cell dysfunction and the immune escape of tumor cells, which is of great importance in tumorigenesis, development, and tumor-targeted therapy. PD-L1 is the most concerned molecule in immunotherapy research. PD-L1 has attracted much attention because of its ability to inhibit T cell function and its adjuvant effect on tumor immune escape. B7-H3 is a promising immune checkpoint molecule that is highly expressed in 74% of NSCLC. B7-H3 can reduce the release of interferon by T cells, weaken the cytotoxic activity of natural killer cells, and play a role in negative immune regulation [1]. Studies in NSCLC have revealed that the dual blockade of B7-H3 and PD-L1 enhances the antitumor reaction compared with a single blocking antibody [11, 14, 40]. That is to say, dual PD-L1 and B7-H3 signaling blockade therapy is a promising treatment strategy for NSCLC.

The analysis of clinical samples showed that the expression of miR-326 in tumor tissues was remarkably lower than that in adjacent tissues, and its expression level was negatively correlated with PD-L1 and B7-H3 (Fig. 2C, D). Furthermore, the negative correlation coefficient between miR-326 and PD-L1/B7-H3 was higher in lymph node metastasis than in situ (Fig. 2E, F), which prompted a possible correlation between miR-326 and metastasis.

Lentivirus was used to overexpress miR-326 in A549 and H1975 cell lines. As expected, PD-L1 and B7-H3 were substantially upregulated with the overexpression of miR-326 (Fig. 3B–F). Similarly, the downregulation of miR-326 in Calu-3 and H1734 cells leads to the decreased expression of PD-L1 and B7-H3 (Supplementary Fig. 2).

B7-H3 and PD-L1 contribute to circumvent CD8^+^ T cell-mediated immune surveillance. Hence, we co-cultured T cells with miR-326 overexpressing tumor cells to detect the levels of the cytokines secreted by T cells and determine the changes in T cell activity. T cells co-cultured with miR-326 overexpressed cells secreted more TNF - α, IFN - γ and IL-2 and less IL-1β, IL-10, and TGF-β. Furthermore, we determine the migration capacity of miR-326 overexpressed cells through transwell assay. The tumor cells that overexpress miR-326 had weakened migration ability (Fig. 4G–I). The co-cultured T cells were dysfunctional and secreted more immunosuppressive factors when miR-326 expression was repressed in H1975 cells (Fig. 5A–F). Moreover, PD-L1 and B7-H3 blocked the negated tumor-promoting efficacy induced by the miR-326 inhibitor; hence, an improved antitumor immunity is mediated by PD-L1/B7-H3.

Finally, we constructed tumor-bearing mice to explore the antitumor effect and immune regulation mechanism of miR-326 in vivo. The IHC staining of tumor tissue showed that enhanced miR-326 expression resulted in increased T cells infiltration. Furthermore, T cells in miR-326 overexpressed tissues secreted more antineoplastic cytokines (TNF-α and IFN-γ) a. CD8^+^ TILs were supposed to determine the metastatic potential in LUAD models. As expected, enhanced miR-326 restrained pulmonary metastasis in tumor-bearing mice. Additionally, inhibition of miR-326 by antagomir largely attenuated antitumor effect of miR-326.

This study confirmed the regulatory role of miR-326 in the expression of key immune checkpoints molecular PD-L1 and B7-H3, and highlighted a promising target for immunotherapy. Notably, miR-326 overexpression partially repressed T cell function by downregulating PD-L1 and B7-H3. This finding highlights the fact that tumor immunity is a complex process regulated by many factors. In addition, PD-L1 and B7-H3 expression is under complex regulation and miR-326 is one of the many contributing factors. Further research is needed for detailed regulatory mechanism.

## Materials and methods

### Cell culture and reagents

A549, H1975, H1734, and Calu-3 and BEAS-2B cell lines were purchased at American Type Culture Collection (Manassas, VA, USA). STR identification and mycoplasma detection were performed. All the cells were cultured in Dulbecco’s modified Eagle’s medium (DMEM, Thermo Fisher Scientific, Waltham, MA, USA, SH30243.01) supplemented with 10% fetal bovine serum (FBS, Thermo Fisher Scientific, Waltham, MA, USA, 10270-106) at 37 °C in an atmosphere containing 5% CO_2_.

### Tissues

Tissues of patients (*n* = 50) were captured during the operation of LUAD. All patients were newly diagnosed in Shenzhen People’s Hospital without any prior treatment. The project has been approved by the Ethics Committee of Shenzhen People’s Hospital. All patients had informed consent.

### Animal experiments

BALB/c mice (male, 6–8 weeks) were obtained from SPF (Beijing, China) Biotechnology Co., Ltd. The mice were bred in an SPF environment and injected subcutaneously with tumors cells (5 × 10^5^ cells in 100 μL of PBS per mouse) when their condition was stable. The mice were randomly divided into four groups with 8 mice in each group to prevent unexpected situations. Tumor size was measured every other day 4 days after the injection. MiR-326 antagomir or negative control (10 nmol) was injected into the tumor every 4 days for 28 days.

### Quantitative real-time polymerase chain reaction (qRT-PCR)

Total RNA was extracted by Trizol (Thermo Fisher Scientific, Waltham, MA, USA, 1559602) according to the manufacture’s protocol. cDNA was generated with 1 μg total RNA using by PrimerScript RT reagent kit with gDNA Eraser (Takara, Kyoto, Japan, RR047A) reverse transcriptase. qRT-PCR was performed using SYBR Green Master Mix (Takara, Kyoto, Japan, RR820A) on an ABI Prism 7500 sequence detector (Applied Biosystems, Foster City, CA, USA) to determine mRNA expression. mRNA and miRNA levels were normalized by GAPDH and U6 sRNA, respectively. The qRT-PCR primers were as follows: GAPDH: ACGGATTTGGTCGTATTGGG (forward) and CGCTCCTGGAAGATGGTGAT (reverse); PD-L1: ACGCATTTACTGTCACGGTTC (forward) and CGGGCCCTCTGTCTGTAGC (reverse); B7-H3: CTCACAGGAAGATGCTGCGT (forward) and CTCTGGGGTGTGATGGTGAC (reverse); ICOSLG: GCAGTTAGAGCCGATCTCCC (forward) and CTGCCTACCATCGCTCTGAC (reverse). Hsa-miR-326 and hsa-U6 were bought from Tiangen (Beijing, China).

### Western blot

The harvested cells were lysed in a lysis buffer (50 mM Tris-HCl (pH 6.8), 2% sodium dodecyl sulfate, 1.5% DL-Dithiothreitol, 10% glycerol and 0.2% Bromophenol blue), and lysed protein was quantified before use. The same amount of lysed protein was loaded for electrophoresis and electrotransfer. The antibody (PD-L1, Cell Signaling Technology, 13684T; B7-H3, Cell Signaling Technology, 14058T; ICOSLG, Novus biological, NBP2-46011;GAPDH, Abcam, ab9485) was incubated according to the concentration indicated in the instruction manual. The expression of protein was detected by enhanced chemiluminescence (Millipore, Burlington, MA, USA) and analyzed by densitometry using Image J. The background correction was done with the value of 50 (called rolling disc in the software). To calculate the relative expression of specific protein, the glyceraldehyde-3-phosphate dehydrogenase (GAPDH) serves as a reference for the sample loading.

### Tumor and T cell co-culture system

Peripheral blood mononuclear cells (PBMCs) from healthy human donors were isolated using Lymphoprep density gradient centrifugation (Stemcell, 07851). PBMCs were cultured in six-well plates at a density of 3 × 10^6^ per well with H1734 or H1975 cell lysate (0.5 mg/ml) and IL-2 (20 ng/ml) for 72 h to promote T cell activation. Stimulated PBMCs were then harvested and purified by Lymphoprep density gradient centrifugation, and co-cultured with the H1734 or H1975 cells at a 10:1 ratio for 16 h. The CD8 positive expressing T cells were purified by CD8 magnetic beads (Miltenyi, 130-096-730). The flow cytometry was then performed to analysis the expression of TNF-α, IFN-γ, IL-10, and IL-1β in CD8^+^ T cells. Co-culture media were assayed for TNF-α, IFN-γ, IL-2, IL-10, IL-1β, and TGF-β using a cytokine ELISA assay.

### Lentivirus infection

The lentivirus used for overexpression and interference was purchased from Genechem (Shanghai, China). Cells (5 × 10^5^) were laid on the six-well plate and incubated for 24 h until the cell density reached about 80% confluence. Lentivirus was added with transfection reagent according to the instruction from the manufacturer. Puromycin (1 μg/mL) was added 72 h after virus addition to screen for stable infection cells. The infection efficiency was verified by qRT-PCR and western blot.

### Transfection of cells with miRNA and inhibitors

MiRNA inhibitors were purchased from Genechem (Shanghai, China). Cells (1.5 × 10^5^) in 1 mL of medium were seeded into 12-well plates and incubated for 24 h. Then, transfection was performed using Lipofectamine 2000 (Thermo Fisher Scientific, Waltham, MA, USA, 52887). The transfected cells were screened using 500 μg/mL G418 (Invitrogen, Carlsbad, CA, USA, 11811023). Transfection efficiency was verified by qRT-PCR and western blots.

### Transwell assay

The cells were resuspended in FBS-free DMEM in the upper transwell chamber at a concentration of 1 × 10^5^ cells/100 μL. Medium (500 μL) containing 20% FBS was added into the bottom chamber as a chemoattractant. After incubation for 24 h, the remaining cells in the inner side of the chamber were removed with cotton swabs. The cells that migrated to the outer side of the chamber membrane were fixed with fixative, stained with 0.5% crystal violet, dried at 80 °C for 30 min, and photographed, Five fields were randomly selected under an inverted microscope to determine the number of cells.

### Flow cytometry

For cell preparation, tumor tissues were dissociated by using according to the instructions, and CD8 T cells were purified by magnetic beads (Miltenyi, 130-045-201). For cultured cells, cells were harvested and adjusted to 1 × 10^5^/100 μl, stained with antibodies (PD-L1-PE, BioLegend, 329705; B7-H3-PE, BioLegend, 331605; CD8-FITC, BioLegend, 344704; IFN-γ-APC, BioLegend, 502511; TNF-α-APC, BioLegend, 502913; IL-10-APC, BioLegend, 501409; IL-1β-APC, 17-7114-80, Thermo; Mouse IgG1-PE, BioLegend, 400112; Mouse IgG1-FITC, BioLegend, 400107; Mouse IgG1-APC, BioLegend, 400119) for 30 min, washed once, and centrifuged at 900 r/min. Finally, the supernatant was removed and resuspended in PBS for flow cytometry. The results of flow cytometry were analyzed using FlowJo software.

### Luciferase activity assay

293T cells in logarithmic growth phase were made into cell suspension, inoculated in a 24-well culture plate, and cultured until the cell fusion reached about 60%. Transfection was carried out with X-tremeGENE HP (Roche) for 48 h. Luciferase was detected using a Dual-Luciferase Reporter Assay System (Promega, Madison, WI, E1960) following a previously established protocol [41, 42].

### Immunohistochemical (IHC) analysis

IHC staining was performed following a previously established protocol [42]. Tumor tissues were fixed in 10% buffered formalin overnight, dehydrated in an ethanol series, cleared in xylene, and embedded in paraffin wax. Sections were cut at 5-μm thickness and stained with CD8 (Abcam, EP1150Y), PD-L1(NOVUS, 13684T), and B7-H3 monoclonal antibodies (Abcam, ab134161). The sections were mounted, viewed, and photographed with a confocal microscope (Leica, Germany).

### MTS assay

Cell growth was measured by 3-(4,5-dimethylthiazol-2yl)-5- (3-carboxymethoxyphenyl)-2-(4-sulphophenyl)-2H-tetrazolium (MTS) assay (Promega) in 96-well plates (1000 cells per well) following the instructions of the manufacturer.

### Enzyme-linked immunosorbent assay (ELISA)

The levels of cytokines were detected using an ELISA kit (Abcam, Cambridge, UK, USA). The experiment was performed following the manufacture’s instruction. Then, enzyme activity was measured at 450 nm using a SPARK 10 M microplate reader (Tecan, Switzerland).

### Bioinformatics analysis

Bioinformatics analysis was performed to predict the putative targets of miR-326 using TargetScan (www.targetscan.org), PicTar (https://pictar.mdc-berlin.de/), and miRBase (http://www.mirbase.org).

### Statistical analysis

Statistical analysis was conducted using GraphPad Prism 5.0 evaluate the differences between different groups. All data are shown as mean ± standard deviation. Comparisons between groups for statistical significance were performed with Student’s *t* test or analysis of variance (ANOVA) with post hoc test in multiple groups. *P* < 0.05 was considered statistically significant.

## Supplementary information

supplemental figure 1

supplemental figure 2

change of authorship request
