# Supplementary figures and images for: MicroRNA-326 attenuates immune escape and prevents metastasis in lung adenocarcinoma by targeting PD-L1 and B7-H3
Source: Cell Death Discov. 2021 Jun 15;7:145. doi: 10.1038/s41420-021-00527-8 (PMC8206349; doi:10.1038/s41420-021-00527-8)

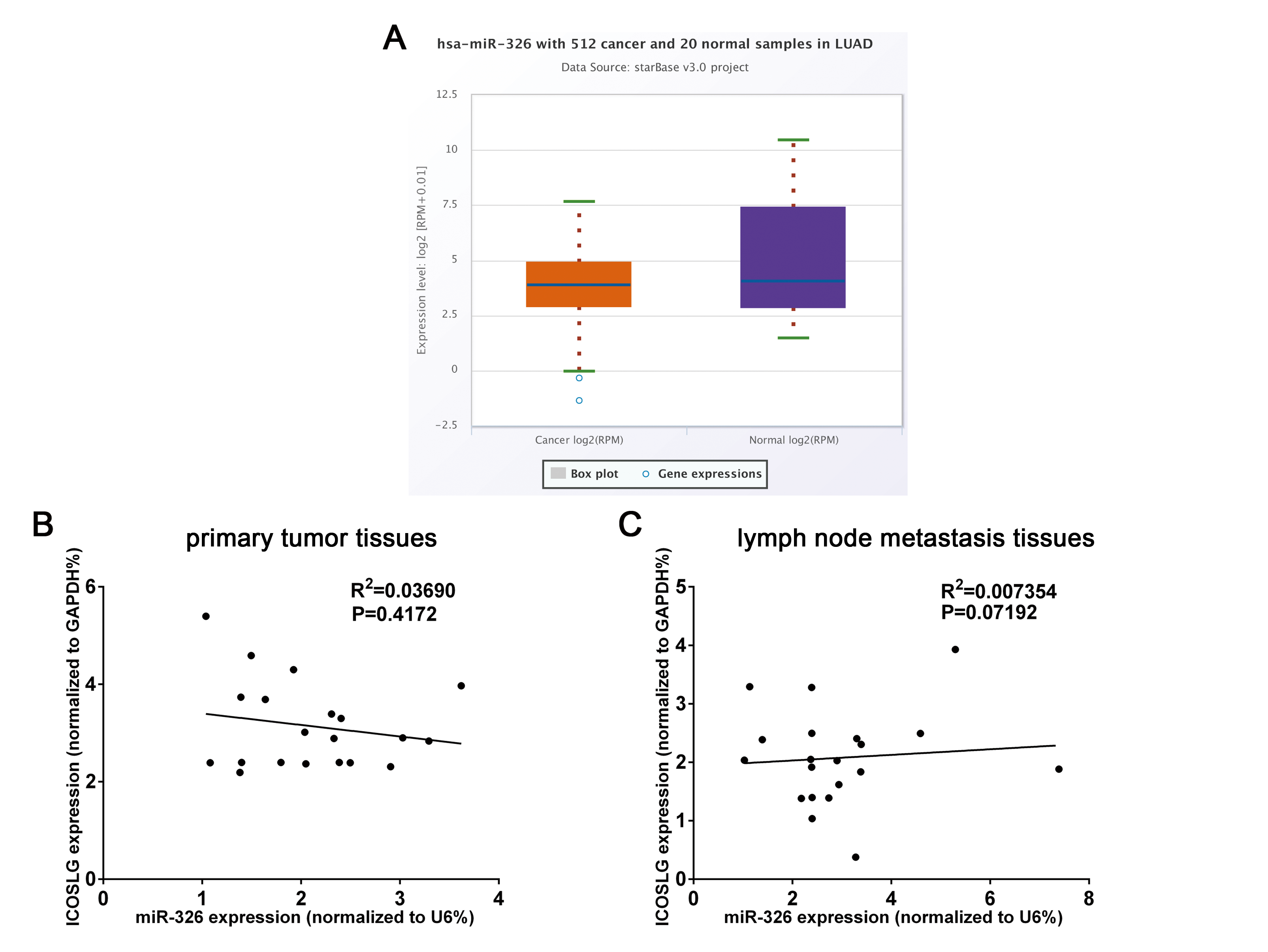

Supplement: Supplementary file 1 — supplemental figure 1 [file 41420_2021_527_MOESM1_ESM.png]

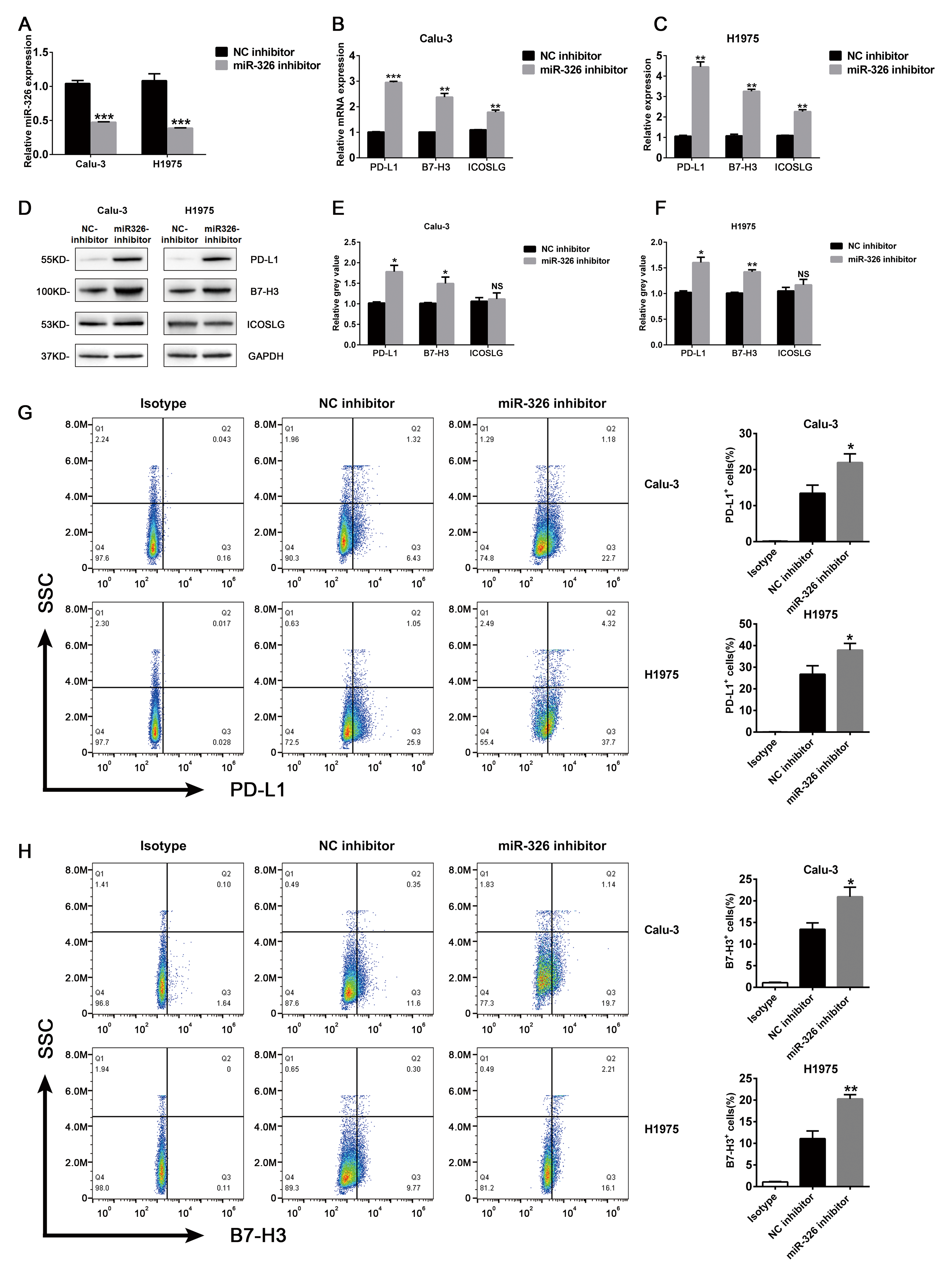

Supplement: Supplementary file 2 — supplemental figure 2 [file 41420_2021_527_MOESM2_ESM.png]
