# Supplementary material for: MicroRNA-326 attenuates immune escape and prevents metastasis in lung adenocarcinoma by targeting PD-L1 and B7-H3
Source: Cell Death Discov. 2021 Jun 15;7:145. doi: 10.1038/s41420-021-00527-8 (PMC8206349; doi:10.1038/s41420-021-00527-8)
Supplement: Supplementary file 3 — change of authorship request [file 41420_2021_527_MOESM3_ESM.pdf]

SPRINGER NATURE

Change of authorship request form - Journals (pre-acceptance)

Important information. Please read.

- This form should be used by authors to request any change in authorship (adding/deleting authors) including changes in corresponding authors. This form should not be used for name changes. Please fully complete all sections. Use black ink and block capitals and provide each author's full name with the given name first followed by the family name.
- By signing this declaration, all authors guarantee that the order of the authors are in accordance with their scientific contribution, if applicable as different conventions apply per discipline, and that only authors have been added who made a meaningful contribution to the work.
- Please note, in author collaborations where there is formal agreement for representing the collaboration, it is sufficient for the representative or legal guarantor (usually the corresponding author) to complete and sign the Authorship Change Form on behalf of all authors, **next to the added/removed author(s)**. **(Complete Section 3, followed by Section 6.)**
  In author collaborations where there is no formal agreement for representing the collaboration and **there are more than 10 authors**, one may sign for all, provided the signer appends correspondence that attests that each of the authors have agreed to the change **and the added/removed authors sign the form**. **(Complete Section 3, followed by Section 6.)**
- Please note, we cannot investigate or mediate any authorship disputes. If you are unable to obtain agreement from all authors (including those who you wish to be removed) you must refer the matter to your institution(s) for investigation. Please inform us if you need to do this.
- If you are not able to return a fully completed form within **30 days** of the date that it was sent to the author requesting the change, we may have to withdraw your manuscript. We cannot publish manuscripts where authorship has not been agreed by all authors (including those who have been removed).
- Incomplete forms will be rejected.
- Please return/upload this form, fully completed, to the Journals Editorial Office. The Journal and/or Publisher will consider the information you have provided to decide whether to approve the proposed change in authorship. We may decide to contact your institution for more information or undertake a further investigation, if appropriate, before making a final decision.

SPRINGER NATURE

Change of authorship request form - Journals (pre-acceptance)

Section 1: Please provide the current title of manuscript

Manuscript ID no.:CDDISCOVERY-21-1410R

Title: MicroRNA-326 attenuates immune escape and prevents metastasis in lung adenocarcinoma by targeting PD-L1 and B7-H3

Section 2: Please provide the previous authorship, in the order shown on the manuscript before the changes were introduced. Please indicate the corresponding author by adding (CA) behind the name.

	First name(s)	Family name	ORCID or SCOPUS id, if available
1st author	Lijuan	Shao	
2nd author	Qian	He	
3rd author	Jingbo	Wang	
4th author	Shengcheng	Lin	
5th author	Liujing	Wu	
6th author	Yubiao	Gao	
7th author	Wei	Ma	
8th author	Jun	Dong	
9th author	Xiaofei	Yang	
10th author	Furong	Li	

Please use an additional sheet if there are more than 10 authors.

SPRINGER NATURE Springer Nature is one of the world's leading global research, educational and professional publishers, created in May 2015 through the combination of Nature Publishing Group, Palgrave Macmillan, Macmillan Education and Springer Science+Business Media.

SPRINGER NATURE

Change of authorship request form - Journals (pre-acceptance)

Section 3: Please provide a justification for change. Please use this section to explain your reasons for changing the authorship of your manuscript, e.g. what necessitated the change in authorship? Please refer to the (journal) policy pages for more information about authorship. Please explain why omitted authors were not originally included and/or why authors were removed on the submitted manuscript.

In the process of revising the paper, Dr. Fei He performed animal experiments and flow cytometry analysis, which contributed to the revision of the paper.

Section 4: Proposed new authorship. Please provide your new authorship list in the order you would like it to appear on the manuscript. Please indicate the corresponding author by adding (CA) behind the name. <u>If the Corresponding Author has changed, please indicate the reason under section 3</u>.

	First name(s)	Family name (this name will appear in full on the final publication and will be searchable in various abstract and indexing databases)	Affiliated institute	E-mail address
1st author	Lijuan	Shao	Shenzhen People's Hospital	shaoshao12071@163.com
2nd author	Qian	He	Shenzhen People's Hospital	angus1216@163.com
3rd author	Jingbo	Wang	Shenzhen People's Hospital	wangjingboqd2008@163.com
4th author	Fei	He	Shenzhen People's Hospital	hf_xmu@163.com
5th author	Shengcheng	Lin	Chinese Academy of Medical Sciences and PeKing Union Medical College	sirius_hzoo@163.com
6th author	Liujing	Wu	Shenzhen People's Hospital	408211372@qq.com
7th author	Yubiao	Gao	Shenzhen People's Hospital	2445602039@qq.com
8th author	Wei	Ma	Shenzhen People's Hospital	364085817@qq.com
9th author	Jun	Dong	Jinan University	dongjunbox@163.com
10th author	Xiaofei	Yang	Shenzhen People's Hospital	sophiayangxf@163.com

Please use an additional sheet if there are more than 10 authors.

SPRINGER NATURE Springer Nature is one of the world's leading global research, educational and professional publishers, created in May 2015 through the combination of Nature Publishing Group, Palgrave Macmillan, Macmillan Education and Springer Science+Business Media.

SPRINGER NATURE

Change of authorship request form - Journals (pre-acceptance)

Section 5: Author contribution, Acknowledgement and Disclosures. Please use this section to provide a new disclosure statement and, if appropriate, acknowledge any contributors who have been removed as authors and ensure you state what contribution any new authors made (if applicable per the journal or book (series) policy). Please ensure these are updated in your manuscript - after approval of the change(s) - as our production department will not transfer the information in this form to your manuscript.

New acknowledgements:

The study was supported by the Science and Technology Project of Shenzhen (No. GJHZ20170310090257380, JCYJ20170413092711058, JCYJ20180228164407689), the Natural Science Foundation of Guangdong Province, China (Grant No. 2018A0303100019), the China Postdoctoral Science Foundation (No.2018M631046).

New Disclosures (financial and non-financial interests, funding):

The authors declare that they have no conflict of interest.

New Author Contributions statement (if applicable per the journal policy):

Lijuan Shao designed the research and finished of the article. Qian He designed the research and interpreted part of the data. Jingbo Wang interpreted the data. Fei He performed animal experiments and flow cytometry analysis. Shengcheng Lin collected pathological tissues and analysed the clinical data. Liujing Wu and Yubiao Gao performed part of the in vitro experiments.Wei Ma performed part of the in vivo experiments. Jun Dong pervaded the technical support. Xiaofei Yang and Furong Li supervised the project.

State 'Not applicable' if there are no new authors.

SPRINGER NATURE Springer Nature is one of the world's leading global research, educational and professional publishers, created in May 2015 through the combination of Nature Publishing Group, Palgrave Macmillan, Macmillan Education and Springer Science+Business Media.

SPRINGER NATURE

Change of authorship request form - Journals (pre-acceptance)

Section 6: Declaration of agreement. ***All*** **authors, unchanged, new and removed** ***must*** **sign this declaration.**
(NB. Please print the form, (docu)-sign and return/upload a scanned copy. Please note that signatures that have been inserted as an image file are acceptable as long as it is handwritten. Typed names in the signature box are unacceptable.) *** Please delete as appropriate. Delete all of the bold if you were on the original authorship list and are remaining as an author.**

	First name	Family name		Signature	Date
1st author	Lijuan	Shao	I agree to the proposed new authorship shown in section 4 /and the proposed change in corresponding author	Lijuan Shao	2021.5.4
2nd author	Qian	He	I agree to the proposed new authorship shown in section 4 /and the proposed change in corresponding author	Qian He	2021.5.4
3rd author	Jingbo	Wang	I agree to the proposed new authorship shown in section 4 /and the proposed change in corresponding author	Jingbo Wang	2021-5-4
4th authors	Fei	He	I agree to the proposed new authorship shown in section 4 **/and the addition/removal*of my name to the authorship list** /and the proposed change in corresponding author	Fei He	2021.5.4
5th author	Shengcheng	Lin	I agree to the proposed new authorship shown in section 4 /and the proposed change in corresponding author	Linshengcheng	2021.5.4
6th author	Liujing	Wu	I agree to the proposed new authorship shown in section 4 /and the proposed change in corresponding author	Liujing Wu	2021.5.4
7th author	Yubiao	Gao	I agree to the proposed new authorship shown in section 4 /and the proposed change in corresponding author	Yubiao Gao	2021.5.4

SPRINGER NATURE Change of authorship request form - Journals (pre-acceptance)

	First name	Family name		Signature	Date
8th author	Wei	Ma	I agree to the proposed new authorship shown in section 4 /and the proposed change in corresponding author	Wei Ma	2021.5.4
9th author	Jun	Dong	I agree to the proposed new authorship shown in section 4 /and the proposed change in corresponding author	Jun Dong	2021.5.4
10th author	Xiaofei	Yang	I agree to the proposed new authorship shown in section 4 /and the proposed change in corresponding author	Yang Xiaofei	2021.5.4

Please use an additional sheet if there are more than 10 authors.

In case of author collaborations with formal agreement:

	Name of consortium/consortia	First name	Family name		Signature	Date
Representative/legal guarantor	Not applicable			I agree to the proposed new authorship shown in section 4 /and the proposed change in corresponding author	Not applicable	

Both added/removed authors should complete the information in the first table under Section 6.

---- End of form ----

SPRINGER NATURE Springer Nature is one of the world's leading global research, educational and professional publishers, created in May 2015 through the combination of Nature Publishing Group, Palgrave Macmillan, Macmillan Education and Springer Science+Business Media.

SPRINGER NATURE

Change of authorship request form - Journals (pre-acceptance)

Important information. Please read.

- This form should be used by authors to request any change in authorship (adding/deleting authors) including changes in corresponding authors. This form should not be used for name changes. Please fully complete all sections. Use black ink and block capitals and provide each author's full name with the given name first followed by the family name.
- By signing this declaration, all authors guarantee that the order of the authors are in accordance with their scientific contribution, if applicable as different conventions apply per discipline, and that only authors have been added who made a meaningful contribution to the work.
- Please note, in author collaborations where there is formal agreement for representing the collaboration, it is sufficient for the representative or legal guarantor (usually the corresponding author) to complete and sign the Authorship Change Form on behalf of all authors, **next to the added/removed author(s)**. **(Complete Section 3, followed by Section 6.)**
  In author collaborations where there is no formal agreement for representing the collaboration and **there are more than 10 authors**, one may sign for all, provided the signer appends correspondence that attests that each of the authors have agreed to the change **and the added/removed authors sign the form**. **(Complete Section 3, followed by Section 6.)**
- Please note, we cannot investigate or mediate any authorship disputes. If you are unable to obtain agreement from all authors (including those who you wish to be removed) you must refer the matter to your institution(s) for investigation. Please inform us if you need to do this.
- If you are not able to return a fully completed form within **30 days** of the date that it was sent to the author requesting the change, we may have to withdraw your manuscript. We cannot publish manuscripts where authorship has not been agreed by all authors (including those who have been removed).
- Incomplete forms will be rejected.
- Please return/upload this form, fully completed, to the Journals Editorial Office. The Journal and/or Publisher will consider the information you have provided to decide whether to approve the proposed change in authorship. We may decide to contact your institution for more information or undertake a further investigation, if appropriate, before making a final decision.

SPRINGER NATURE

Change of authorship request form - Journals (pre-acceptance)

Section 1: Please provide the current title of manuscript

Manuscript ID no.:CDDISCOVERY-21-1410R

Title: MicroRNA-326 attenuates immune escape and prevents metastasis in lung adenocarcinoma by targeting PD-L1 and B7-H3

Section 2: Please provide the previous authorship, in the order shown on the manuscript before the changes were introduced. Please indicate the corresponding author by adding (CA) behind the name.

	First name(s)	Family name	ORCID or SCOPUS id, if available
1st author	Lijuan	Shao	
2nd author	Qian	He	
3rd author	Jingbo	Wang	
4th author	Shengcheng	Lin	
5th author	Liujing	Wu	
6th author	Yubiao	Gao	
7th author	Wei	Ma	
8th author	Jun	Dong	
9th author	Xiaofei	Yang	
10th author	Furong	Li	

Please use an additional sheet if there are more than 10 authors.

SPRINGER NATURE Springer Nature is one of the world's leading global research, educational and professional publishers, created in May 2015 through the combination of Nature Publishing Group, Palgrave Macmillan, Macmillan Education and Springer Science+Business Media

SPRINGER NATURE Change of authorship request form - Journals (pre-acceptance)

Section 3: Please provide a justification for change. Please use this section to explain your reasons for changing the authorship of your manuscript, e.g. what necessitated the change in authorship? Please refer to the (journal) policy pages for more information about authorship. Please explain why omitted authors were not originally included and/or why authors were removed on the submitted manuscript.

In the process of revising the paper, Dr. Fei He performed animal experiments and flow cytometry analysis, which contributed to the revision of the paper.

Section 4: Proposed new authorship. Please provide your new authorship list in the order you would like it to appear on the manuscript. Please indicate the corresponding author by adding (CA) behind the name. If the Corresponding Author has changed, please indicate the reason under section 3.

	First name(s)	Family name (this name will appear in full on the final publication and will be searchable in various abstract and indexing databases)	Affiliated institute	E-mail address
11th author	Furong	Li (CA)	Shenzhen People's Hospital	frli62@163.com

Please use an additional sheet if there are more than 10 authors.

SPRINGER NATURE Springer Nature is one of the world's leading global research, educational and professional publishers, created in May 2015 through the combination of Nature Publishing Group, Palgrave Macmillan, Macmillan Education and Springer Science+Business Media.

SPRINGER NATURE

Change of authorship request form - Journals (pre-acceptance)

Section 5: Author contribution, Acknowledgement and Disclosures. Please use this section to provide a new disclosure statement and, if appropriate, acknowledge any contributors who have been removed as authors and ensure you state what contribution any new authors made (if applicable per the journal or book (series) policy). Please ensure these are updated in your manuscript - after approval of the change(s) - as our production department will not transfer the information in this form to your manuscript.

New acknowledgements:

The study was supported by the Science and Technology Project of Shenzhen (No. GJHZ20170310090257380, JCYJ20170413092711058, JCYJ20180228164407689), the Natural Science Foundation of Guangdong Province, China (Grant No. 2018A0303100019), the China Postdoctoral Science Foundation (No.2018M631046).

New Disclosures (financial and non-financial interests, funding):

The authors declare that they have no conflict of interest.

New Author Contributions statement (if applicable per the journal policy):

Lijuan Shao designed the research and finished of the article. Qian He designed the research and interpreted part of the data. Jingbo Wang interpreted the data. Fei He performed animal experiments and flow cytometry analysis. Shengcheng Lin collected pathological tissues and analysed the clinical data. Liujing Wu and Yubiao Gao performed part of the in vitro experiments.Wei Ma performed part of the in vivo experiments. Jun Dong pervaded the technical support. Xiaofei Yang and Furong Li supervised the project.

State 'Not applicable' if there are no new authors.

SPRINGER NATURE Springer Nature is one of the world's leading global research, educational and professional publishers, created in May 2015 through the combination of Nature Publishing Group, Palgrave Macmillan, Macmillan Education and Springer Science+Business Media.

SPRINGER NATURE **Change of authorship request form - Journals** (pre-acceptance)

Section 6: Declaration of agreement. ***All*** **authors, unchanged, new and removed** ***must*** **sign this declaration.**

(NB: Please print the form, (docu)-sign and return/upload a scanned copy. Please note that signatures that have been inserted as an image file are acceptable as long as it is handwritten. Typed names in the signature box are unacceptable.) *** Please delete as appropriate. Delete all of the bold if you were on the original authorship list and are remaining as an author.**

	First name	Family name		Signature	Date
11th author	Furong	Li(CA)	I agree to the proposed new authorship shown in section 4 /and the proposed change in corresponding author	Furong Li	2021.5.4

SPRINGER NATURE Springer Nature is one of the world's leading global research, educational and professional publishers, created in May 2015 through the combination of Nature Publishing Group, Palgrave Macmillan, Macmillan Education and Springer Science+Business Media.

SPRINGER NATURE

Change of authorship request form - Journals (pre-acceptance)

	First name	Family name		Signature	Date

Please use an additional sheet if there are more than 10 authors.

In case of author collaborations with formal agreement:

	Name of consortium/consortia	First name	Family name		Signature	Date
Representative/legal guarantor	Not applicable			I agree to the proposed new authorship shown in section 4 /and the proposed change in corresponding author	Not applicable	

Both added/removed authors should complete the information in the first table under Section 6.

---- End of form ----
